# Risk of Respiratory Syncytial Virus Hospitalization in the First and Second Years of Life in Pediatric Patients with Congenital Heart Disease

**DOI:** 10.1007/s00246-017-1634-5

**Published:** 2017-05-23

**Authors:** Deborah Friedman, Pierre C. Wong

**Affiliations:** 1 Department of Pediatrics, New York Medical College, Valhalla, NY USA; 2 Division of Cardiology, Children’s Hospital, Los Angeles, CA USA

Previous data have demonstrated that children aged <24 months with hemodynamically significant congenital heart disease (HS-CHD) are at elevated risk for severe respiratory syncytial virus (RSV) disease. However, based upon the more recent assertion that children aged 12–23 months are not at increased risk of severe RSV, the American Academy of Pediatrics Committee on Infectious Diseases altered its guidance in 2014, recommending against RSV immunoprophylaxis in children with CHD in the second year of life [1].

Although children aged 12–23 months with CHD might have an overall lower risk of RSV hospitalization (RSVH) compared with children aged <12 months, given the heterogeneity of CHD, specific CHD diagnoses can still be associated with RSVH of greater severity. We have recently shown that certain forms of HS-CHD are associated with high-severity RSVH in children aged 12–23 months [2]. Here we provide similar data for children aged 0–11 months.

We evaluated the impact of RSVH on mortality, mechanical ventilation (MV), and hospital charges in children aged 0–11 and 12–23 months with specific HS-CHD diagnoses. We identified patients aged <2 years with RSVH from the 1997–2013 National Inpatient Sample of US inpatient admissions, and compared patients with CHD to age-matched cohorts without CHD.

During the 17-year study period, 35,634 RSVHs were identified in CHD patients aged 0–11 months, with 1.2% mortality, 19% MV, and mean charges of $23,972. Diagnoses with the highest severity (mortality ≥ 4%) included cardiomyopathy, heterotaxia, total anomalous pulmonary venous return, aortic stenosis, and CHF. Over the same period, RSVHs for age-matched non-CHD patients had 0.1% mortality, 3.8% MV, and mean charges of $8488.

For CHD patients aged 12–23 months, 4813 RSVHs were identified, with 1.5% mortality, 12% MV, and mean charges of $19,650. The highest-severity CHD diagnoses (mortality > 6%) included transposition of the great vessels, cardiomyopathy, CHF, Ebstein’s anomaly, and aortic stenosis. Age-matched non-CHD patients had 0.1% mortality, 2.3% MV, and mean charges of $8000.

We conclude that in the first 24 months of life, children with specific high-risk forms of CHD that are hospitalized for RSV have a significantly increased risk of morbidity/mortality and increased hospital charges compared to children without CHD. The risk is substantially increased during the first year of life, but it continues into the second year of life, particularly with the above-identified CHD diagnoses (including transposition of the great vessels, cardiomyopathy, CHF, Ebstein’s anomaly, aortic stenosis). The data from this study reinforce the continuing need for RSV prevention in infants and children with HS-CHD, even into the second year of life.
